# *Plectranthus* Species with Anti-Inflammatory and Analgesic Potential: A Systematic Review on Ethnobotanical and Pharmacological Findings

**DOI:** 10.3390/molecules28155653

**Published:** 2023-07-26

**Authors:** Maysa de Oliveira Barbosa, Polrat Wilairatana, Giovana Mendes de Lacerda Leite, Gyllyandeson de Araújo Delmondes, Lucas Yure Santos da Silva, Silvio Caetano Alves Júnior, Lindaiane Bezerra Rodrigues Dantas, Daniel Souza Bezerra, Izabel Cristina Santiago Lemos de Beltrão, Diógenes de Queiroz Dias, Jaime Ribeiro-Filho, Cícero Francisco Bezerra Felipe, Henrique Douglas Melo Coutinho, Irwin Rose Alencar de Menezes, Marta Regina Kerntopf Mendonça

**Affiliations:** 1 Department of Biological Chemistry, Regional University of Cariri-URCA, Crato 63105-000, CE, Brazil; maysabarbosa.ce@gmail.com (M.d.O.B.); giovanalacerda_@hotmail.com (G.M.d.L.L.); lucas.yure@urca.br (L.Y.S.d.S.); lindaianebrd@gmail.com (L.B.R.D.); danielbezerra02@gmail.com (D.S.B.); izabel.lemos@urca.br (I.C.S.L.d.B.); diogenes@gmail.com (D.d.Q.D.); hdmcoutinho@gmail.com (H.D.M.C.); martaluiz@yahoo.com.br (M.R.K.M.); 2 Department of Clinical Tropical Medicine, Faculty of Tropical Medicine, Mahidol University, Bangkok 10400, Thailand; 3 Collegiate of Nursing, Federal University of Vale do São Francisco (UNIVASF), Petrolina 56304-917, PE, Brazil; gyllyandeson.delmondes@univasf.edu.br; 4 Oswaldo Cruz Foundation (FIOCRUZ), Fiocruz Ceará, Eusébio 61773-270, CE, Brazil; silvio.alves.bio@gmail.com (S.C.A.J.); jaime.ribeiro@fiocruz.br (J.R.-F.); 5 Department of Molecular Biology, Federal University of Paraiba, João Pessoa 58051-900, PB, Brazil; cicero@dbm.ufpb.br

**Keywords:** *Plectranthus*, inflammation, nociception, ethnobotany, ethnopharmacology, pharmacological trials, systematic review

## Abstract

The use of medicinal plants to treat inflammatory conditions and painful processes has attracted the attention of scientists and health professionals due to the evidence that natural products can promote significant therapeutic benefits associated with fewer adverse effects compared to conventional anti-inflammatory drugs. The genus *Plectranthus* is composed of various plants with pharmacological potential, which are used to treat various diseases in traditional communities worldwide. The present study systematically reviewed *Plectranthus* species with anti-inflammatory and analgesic potential. To this end, a systematic review was conducted following the Preferred Reporting Items for Systematic Reviews and Meta-Analyses (PRISMA) protocol. The search was conducted on the following databases: PubMed, ScienceDirect, SciVerse Scopus, and Web of Science. Different combinations of search terms were used to ensure more excellent article coverage. After the selection, a total of 45 articles were included in this review. This study identified twelve *Plectranthus* species indicated for the treatment of different inflammatory conditions, such as wounds, fever, bronchitis, abscess, asthma, hepatitis, labyrinthitis, tonsillitis, and uterine inflammation. The indications for pain conditions included headache, sore throat, heartburn, menstrual cramp, colic, toothache, stomachache, migraine, chest pain, abdominal pain, local pain, labor pain, and recurring pain. Among the listed species, ten plants were found to be used according to traditional knowledge, although only four of them have been experimentally studied. When assessing the methodological quality of preclinical in vivo assays, most items presented a risk of bias. The SR results revealed the existence of different *Plectranthus* species used to treat inflammation and pain. The results of this systematic review indicate that *Plectranthus* species have the potential to be used in the treatment of diseases with an inflammatory component, as well as in the management of pain. However, given the risk of biases, the experimental analysis of these species through preclinical testing is crucial for their safe and effective use.

## 1. Introduction

Ethnobotany and ethnopharmacology investigate the connection between plants and humans through a therapeutic point of view, investigating how traditional medical practices can contribute to exploring new therapeutic compounds [1]. In addition to preserving biodiversity-based therapeutic practices, traditional medicine has significantly contributed to scientific advancement in diverse investigation fields [2]. Notably, the organization of this knowledge through systematic reviews has significantly impacted drug discovery [3]. By synthesizing and analyzing previously reported findings, systematic reviews provide a comprehensive and trustworthy assessment of the current research landscape, offering a more robust understanding of specific issues [4]. Preclinical research has experimentally confirmed the therapeutic potential of plants, herbal remedies, and isolated compounds reported in traditional knowledge sources for the treatment of pain and inflammation. In addition, preclinical research has contributed to understanding the mechanisms of action and potential clinical applications of anti-inflammatory and analgesic natural products [5].

Inflammatory diseases and painful conditions are managed with different drug classes. In this context, nonsteroidal anti-inflammatory drugs (NSAIDs), opioids, and corticosteroids are widely prescribed and used worldwide [6]. However, especially in the long term, these drugs are associated with significant adverse effects, including renal impairment, gastritis and gastric ulcers, platelet dysfunction, hemorrhages, dependence, and psychiatric effects such as depression and psychosis. In addition, many of these drugs can cause immunological and nonimmunological hypersensitivity reactions such as anaphylactic reactions, urticaria, and various late cutaneous and organ-specific reactions [7].

The genus *Plectranthus* (Lamiaceae) comprises a wide variety of plants with global distribution and significant pharmacological potential. In addition, species of this genus have been used for ornamental and economic purposes [8]. Although approximately 300 *Plectranthus* species have been identified, only 62 species had their medicinal use investigated. In this context, evidence has indicated that these species have analgesic, anti-inflammatory, antibacterial, and anti-ulcer properties. *Plectranthus* species are promising sources of essential oil and their biologically active constituents, including monoterpenes, diterpenes, and sesquiterpenes. Additionally, 100 other organic compounds of different classes, such as flavonoids, alkaloids, and tannins, have been identified in this genus, many of which have had their pharmacological properties demonstrated [9,10,11].

The present review aims to integrate ethnobotanical, phytochemical, and pharmacological research findings involving *Plectranthus* species through a systematic review meta-analysis. This study intends to contribute to understanding this genus’s therapeutic applications to guide future research on anti-inflammatory and analgesic drug development.

## 2. Results and Discussion

### 2.1. Selecting the Sources of Information

**Figure 1:**
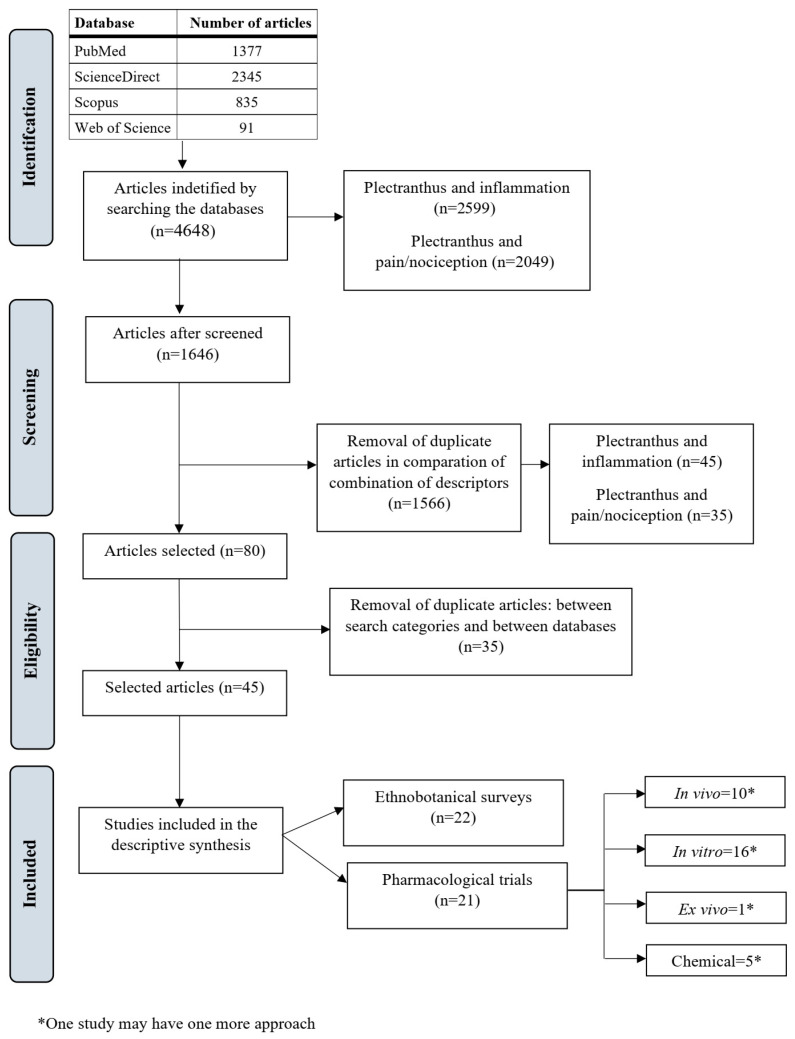
The flowchart diagram describing step by step the articles analysis process of eligible studies included in this systematic review.

The initial search using all combinations of keywords found 4648, with 2599 reporting *Plectranthus* and inflammation and 2049 to *Plectranthus* and pain/nociception (Figure 1). Details on the article search are shown in Table S1. After abstract reading and duplicate exclusion, a total of 43 articles (Figure 1) were included in this review, 22 (51.16%) of which were focused on ethnobotanical surveys, while 21 (48.84%) of them consisted of pharmacological trials.

**Figure 2:**
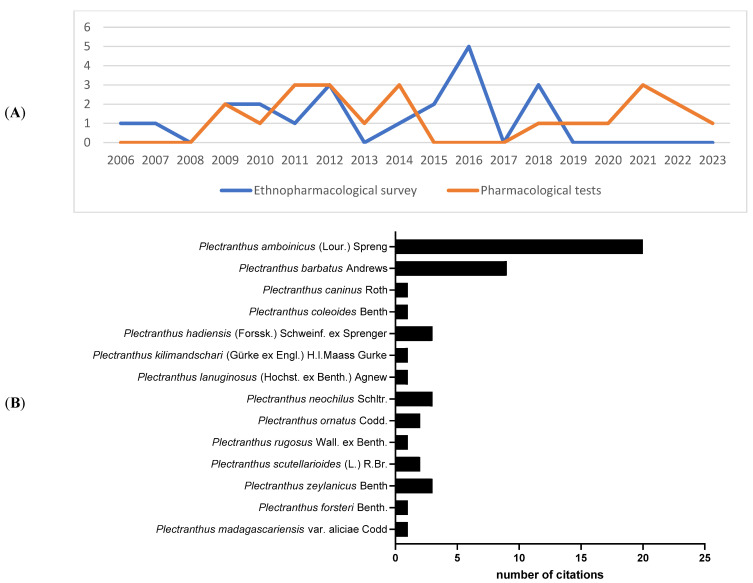
Ethnobotanical surveys data and pharmacological trials: distribution of articles over the years (**A**) and total number of citations of each eligible *Plectranthus* species (**B**) included in this systematic review.

The review identified fourteen species of *Plectranthus* and showed that most studies were published in 2012 (Figure 2A). As shown in Figure 2B, *Plectranthus amboinicus* was the most frequently mentioned species (20 mentions), possibly reflecting its relevance in traditional medicine.

### 2.2. Ethnobotanical Studies

Table 1 presents information on the use of *Plectranthus* species according to ethnobotanical studies. It was observed that most studies were carried out in Africa, South America, and Asia, reflecting the relevance of the genus in the traditional medicine of these continents. Evidence indicates that these areas, along with Oceania (Australia), are the primary habitat for *Plectranthus* species [12,13].

Some species of this genus were introduced and cultivated in these countries due to favorable climatic conditions. The medicinal use of this genus is of particular importance in South America. The genus was introduced in Brazil during the 16th century, at the beginning of the colonial period. In this country, the extensive use of *Plectranthus* species to treat pain and inflammation is related to easy access to plants, in contrast to the difficult access to health services and medicines [14]. Notably, out of the 250,000 species cataloged by the United Nations Educational, Scientific and Cultural Organization (UNESCO), 20% are native to Brazil, which favors their use in managing diseases by local communities [11].

Ten species were reported in ethnobotanical studies: *Plectranthus amboinicus* (Lour.) Spreng [15,16,17,18,19,20,21,22,23], *Plectranthus barbatus* (Andrews) Benth. [15,18,24,25,26,27,28,29], *Plectranthus neochilus* Schtr. [15,30], *Plectranthus coleoides* Benth. [31], *Plectranthus kilimandschari* Gurke [32], *Plectranthus lanuginosus* [33], *Plectranthus ornatus* Codd. [18,21], *Plectranthus rugosus* [34], *Plectranthus scutellarioides* (L.) R. Br. [35] and *Plectranthus zeylanicus* Benth. [36]. 

*Plectranthus amboinicus* (Lour.) Spreng receives a variety of popular names such as “hortelã-folha” [15,24], “malva-do-reino” [17,19], “orégano Cubano” [18], “pêng pèng xiāng” [23], “oregano” [20], “malvarisco” [21], and “omavalli” [22].

*Plectranthus amboinicus* (Lour.) Spreng is a species of African origin, primarily found in the eastern and southeastern regions of the continent, where a tropical climate prevails [37]. Its distribution in the Americas extends from the Antilles region to southern Brazil [38]. *Plectranthus aromaticus* Roxb., *Coleus aromaticus* Benth., and *Coleus amboinicus* Lour. are considered synonyms of *P. amboinicus* [39]. This plant is known to be a widely versatile natural resource. In addition to its application in traditional medicine, its aromatic leaves and refreshing scent are used in gastronomy to flavor various dishes, especially meats [40].

While the leaves were the part of the plant mainly used [18,20,21,22,23,25,26,27,31,32,41], the roots [36] and the whole plant [29] were also mentioned. However, 8 of the 21 studies did not provide information on the part of the plant used [15,16,17,28,30,33,35]. According to the literature, various plant components of *the Plectranthus* species can be considered for medical use, including the leaves, stem, roots, and tubers [42].

The leaves are often used in folk medicine due to their medicinal properties and accessible collection and preparation method. They also contain various chemical compounds with antioxidant, anti-inflammatory, analgesic, and antimicrobial properties. From the perspective of natural resource conservation, the predominant use of leaves in medicinal preparations is positive as it does not cause the death of the collected specimen, thus contributing to the preservation of the local flora [43,44,45] The decoction technique was the most used form of preparation [17,18,20,23,36], followed by infusion [15,27,32,36,41] and maceration [15,18]. Syrup [17,19], juice [17,22,31,41], and leaf paste [31,34] were also reported. However, only one study details the plant preparation process [22]. Regarding the administration route, the oral route was the most reported [17,20,21,25,31,35], corroborating the frequent use of teas (infusions) in folk medicine [46].

**Table 1:** Main study aspects of ethnobotanical surveys.

Author, Year	Place	Country	Cited Species	Use in Inflammation	Use in Pain	Pharmaceutical Form	Part Used	Preparation	Administration	Total of Informants
Ignacimuthu et al., 2006 [31]	Madurai, Tamil Nadu	India	*P. coleoides* Benth.	Wound healing	Labor pain (during pregnancy)	Juice, paste of leaves	Leaves	NR	Oral (drink), local administration	12
Maregesi et al., 2007 [32]	Bunda	Tanzânia	*P. kilimandschari* Gurke		Chest pain	Infusion	Leaves	NR	NR	10
Ferreira, 2009 [25]	Marudá, Pará	Brazil	*P. barbatus* (Andrews) Benth.	Fever	Nonspecific pain, toothache	Fresh infusion,	Leaves	NR	Oral	37
Pereira et al., 2009 [28]	Ponta Porã, Mato Grosso	Brazil	*P. barbatus* (Andrews) Benth.	-	Recurrent pain	NR	NR	NR	NR	137
Cartaxo et al., 2010 [17]	Riacho Catingueira, Aiuaba, Ceará	Brazil	*P. amboinicus* (Lour.) Spreng	Bronchitis, uterine inflammation, inflammation of internal organs, nonspecific inflammation	Headache	Decoction, syrup, juice	NR	NR	Oral (drink) or bathing	91
Rahmatullah et al., 2010 [29]	Khulna	Bangladesh	*P. barbatus* (Andrews) Benth.	-	Cramps	NR	Whole plant	NR	NR	NR
Waruruai et al., 2011 [35]	Bougainville	Papua New Guinea	*P. scutellarioides* (L.) R. Br.	-	Headache	NR	NR	NR	Oral	21
Bieski et al., 2012 [15]	Pantanal, Mato Grosso	Brazil	*P. amboinicus* (Lour.) Spreng	Bronchitis, uterine inflammation	-	Infusion	NR	NR	NR	262
			*P. barbatus* (Andrews) Benth.	-	Pain	Maceration	NR	NR	NR	
			*P. neochilus* Schtr.	Labyrinthitis	Pain	Maceration	NR	NR	NR	
Furlanetto et al., 2012 [18]	Mandaguaçu, Paraná	Brazil	*P. amboinicus* (Lour.) Spreng	Gastritis	Headache	Maceration, decoction	Leaves	NR	NR	220
			*P. barbatus* (Andrews) Benth.	Gastritis	Headache	Maceration, decoction	Leaves	NR	NR	
			*P. ornatos* Codd.	Gastritis	Headache	Maceration, decoction	Leaves	NR	NR	
Ong and Kim, 2014 [20]	Ati Negrito, Guimaras	Filipinas	*P. amboinicus* (Lour.) Spreng	Asthma	-	Decoction	Leaves	NR	Oral	65
Bieski et al., 2015 [16]	Vale do Juruena, Legal Amazon, Mato Grosso	Brazil	*P. amboinicus* (Lour.) Spreng	Wound healing, fever, gastritis	Local pain	NR	NR	NR	NR	383
			*P. barbatus* (Andrews) Benth.	Fever, labyrinthitis	Heartburn, pain, local pain, menstrual cramps	NR	NR	NR	NR	
Oliveira et al., 2015 [26]	Oriximiná, Pará	Brazil	*P. barbatus* (Andrews) Benth.		Migraine	NR	Leaves	NR	NR	35
Lemos et al., 2016 [19]	Barbalha, Ceará	Brazil	*P. amboinicus* (Lour.) Spreng	Bronchitis	Sore throat	Infusion, juice, syrup	Leaves	NR	NR	54
Li and Xing, 2016 [23]	Hainan	China	*P. amboinicus* (Lour.) Spreng	Abscess	Pain	Decoction	Leaves	NR	NR	27
Pedrollo et al., 2016 [21]	Jauaperi, Roraima	Brazil	*P. amboinicus* (Lour.) Spreng	-	Headache	NR	Leaves	NR	Oral	62
			*P. ornatus* Codd.	-	Bellyache	NR	Leaves	NR	Oral	
Santana et al., 2016 [30]	Quilombo Salamina Putumujumar, Bahia	Brazil	*P. neochilus* Schtr.	-	Cramps	NR	NR	NR	NR	74
Penido et al., 2016 [27]	Imperatriz, Maranhão	Brazil	*P. barbatus* (Andrews) Benth.	Hepatite	Stomachache	Infusion	Leaves	NR	NR	205
Rajalakshmi et al., 2019 [22]	Thanjavur, Tamil Nadu	Índia	*P. amboinicus* (Lour.) Spreng	–	Headache	Juice	Leaves	10 g of leaves with sesame oil	Topical use	137
Napagoda et al., 2018 [36]	Gampaha	Sri Lanka	*P. zeylanicus* Benth.	Fever	-	Decoction, infusion	Roots	NR	NR	458
Kidane et al., 2018 [33]	Ganta Afeshum, Tigray	Ethiopia	*P. lanuginosus*	Tonsillitis	-	NR	NR	NR	NR	78

NR = not reported.

Table 2 shows the number and relative frequency of citations (RFC) of *Plectranthus* species in ethnobotanical studies reporting their use in the treatment of inflammation and pain. Higher RFC values indicate a higher level of data homogeneity, considering the versatility of pharmaco-therapeutic properties or observed toxicity effects. It was observed that *Plectranthus amboinicus* and *Plectranthus barbatus* are the most representative species of this genus, with a relatively uniform distribution and remarkable consensus in their citation. Despite the significant variation in their chemical constituents, these species are considered efficient in treating pain and inflammation.

The indications of *Plectranthus* species for painful processes included headache, sore throat, heartburn, menstrual cramps, colic, toothache, stomachache, migraine, chest pain, abdominal pain, local pain, nonspecific pain, labor pain, and recurring pain. Among these, headache was the most frequently reported [17,18,21,22,35]. Projections indicate that 99% of women and 95% of men will have cephalalgia (the medical term for headache) at least once in their lifetime. The data also show that 40% of these people feel or will feel it with a certain periodicity [47].

Regarding inflammation, the species were indicated for treating wounds, fever, bronchitis, uterine inflammation, abscess, asthma, hepatitis, labyrinthitis, tonsillitis, inflammation of internal organs, and nonspecific inflammation. *Plectranthus* species were mainly indicated in this context due to their wound healing properties. Since prehistoric times, plants have been used for wound care, where they could be applied directly to the injury through poultices to stop bleeding and accelerate the healing process or ingested to act systemically [48,49].

In a comprehensive review study on the ethnobotanical uses of this genus, around 20 species of *Plectranthus* were indicated for skin-related conditions, including wound healing. In comparison, 21 species were indicated for digestive disorders. Additionally, 15 types of *Plectranthus* were reported to treat fever [39], corroborating the present findings.

In this study, five species were simultaneously indicated for treating pain and inflammation: *P. amboinicus* (Lour.) Spreng. [16,17,18,23,41], *P. barbatus* (Andrews) Benth. [16,18,25,27], *P. neochilus* Schtr. [15], *P. coleoides* Benth. [31] and *P. ornatus* Codd. [18]. Researchers claim that *Plectranthus* species have the potential to be used in the treatment of fever, pain, skin diseases, respiratory and genitourinary infections, and musculoskeletal, circulatory, and blood disorders, among others [39,50].

### 2.3. Pharmacological Studies

A detailed synthesis of the 22 experimental studies included in this review was achieved by presenting their main findings, as shown in Table 3. Ten species were investigated: *Plectranthus aliciae* [51], *Plectranthus amboinicus* (Lour.) Spreng [40,52,53,54,55,56,57,58,59,60,61], *Plectranthus barbatus* (*Andrews*) Benth [62], *Plectranthus* caninus Roth [63], *Plectranthus forsteri* [64], *Plectranthus hadiensis* (*Hribera*) [65,66,67], *Plectranthus neochilus* [68], *Plectranthus scutellarioides* (L.) *R. Br.* [69], and *Plectranthus zeylanicus* Benth. [70,71].

The ethanolic extract of the species *P. aliciae* and its constituent, rosmarinic acid, were encapsulated in gold nanoparticles and tested for antibacterial effects against aerobic and anaerobic bacteria present in epidermal acne vulgaris (*Cutibacterium acnes* and *Staphylococcus epidermis*). Although the compounds showed low toxicity to human keratinocytes and were effective in treating skin wounds, no antibacterial activity or inhibition of the biofilm was observed. Gold nanoparticles containing rosmarinic acid (29.2 g/mL *v*/*v*) were found to significantly increase wound closure by 21.4% to 25% compared to negative cellular control and pure rosmarinic acid at the highest tested concentration (500 g/mL) [51]. This study shows that encapsulating the main compound of *P. aliciae*, rosmarinic acid, has significant healing effects.

In vivo and in vitro research demonstrated that *P. amboinicus* presented significant anti-inflammatory, analgesic, antimicrobial, antioxidant, and antitumor activities and protected against metabolic disorders. Two species had the essential oil evaluated, where carvacrol was found as the principal constituent. In the studies evaluating the activity of extracts, rosmarinic acid was the most significant secondary metabolite identified in the chemical analyses.

The aqueous extract of *P. amboinicus* leaves significantly decreased paw edema in rats with collagen-induced arthritis, which was associated with reduced levels of IgM, anti-collagen CRP, and pro-inflammatory cytokines such as tumor necrosis factor-alpha (TNF-α), interleukin-6 (IL-6) and interleukin-1-beta (IL-1β) [52]. In RA, elevated levels of these cytokines activate synovial mesenchymal cells and increase the production of prostaglandins and metalloproteinases. It was suggested that the anti-inflammatory effects of this species were due to the presence of thymoquinone, identified in the hexane extract of *P. amboinicus* [42]. Notably, the quinone group, present in compounds of several species of Lamiaceae, besides presenting anti-inflammatory activity, has antibacterial, antihypertensive, antidiabetic, neuroprotective, anti-apoptotic, and apoptotic effects [72].

The study of [40] sought to investigate the constituents of the aqueous and hexane extract of *P. amboinicus* and prepare analogs with therapeutic potential for treating rheumatoid arthritis. They showed that 2-(3,4-dihydroxybenzylidenyl)-3-(3,4-hydroxyphenyl)-4-hydroxy-pentane dioic acid, shimobashyric acid, salvianolic acid, and rosmarinic acid inhibited the binding of the transcription factor AP-1 to its consensual DNA sequence.

Disease-modifying antirheumatic drugs that block cytokine signaling are promising therapeutic agents in rheumatoid arthritis, targeting disease-related biological factors such as TNF-α and transcription factor AP-1. Therefore, the study shows that the constituents of the *P. amboinicus* species and their analogs may significantly affect arthritis, a progressive [52,56] chronic disease. These results are further evidenced by the studies conducted by [52,56].

The study of [56] evaluated the inhibitory effects of osteoclastogenesis and inflammatory bone erosion of *P. amboinicus* in mice with collagen-induced arthritis (AIC). The authors found that the extract of this species considerably inhibited bone resorption activity of mature osteoclasts at a dose of 375 mg/kg. A study by [55] showed that the equivalent dose (350 mg/kg) also presented a significant antiedematogenic effect in rats’ paw edema model induced by carrageenan. These authors also reported the inhibitory effects of the extract in the growth of sarcoma-180 and Ehrlich’s ascites tumors, considering the doses of 100, 150, 250, and 350 mg/kg.

The aqueous extract and essential oil of *P. amboinicus* showed analgesic and anti-inflammatory activities. It was demonstrated that its mechanism is related to the modulation of antioxidant enzymatic activities in the liver and the decrease in malondialdehyde (MDA), tumor necrosis factor-alpha (TNF-α), and cyclooxygenase2 (COX-2). Through in vitro assays, these authors observed inhibitory effects on lipopolysaccharide (LPS)-stimulated RAW 264.7 cells that were associated with the degradation of IκB-α and nuclear translocation of the p65 subunit of NF-Κb [53].

The work of [57] also evaluated the anti-inflammatory activity of aqueous extracts and ethyl acetate of *P. amboinicus*, demonstrating that the expression of oxidative stress markers, iNOS, COX-2, IL-1β, histamine receptor 1, and NF-Κb was modulated by the pretreatment with the extracts. In addition, the same treatments resulted in decreased NO production, indicating inhibition of macrophage activation. Studies investigating the anti-inflammatory mechanisms of *P. amboinicus* (*PA-F4*) demonstrated the inhibition of the NLRP3 inflammasome. PA-F4 inhibited the ATP-induced release of caspase-1, IL-1β, and IL-18 from lipopolysaccharide-initiated cells (LPS) by blocking NF-kB activation. These authors suggested that rosmarinic acid, cirsimaritin, salvigenin, and carvacrol are the active components of the extract [59]. The study of [56] showed that rosmarinic acid inhibited the activation of the transcription factor NF-κB and NFATc1 in bone marrow macrophages (BMM). Moreover, evidence indicates that *P. amboinicus* has anti-inflammatory, antibacterial, and antifungal activities partially mediated by carvacrol [60]. These data corroborate the previously mentioned studies and demonstrate the therapeutic potential of *P. amboinicus* in inflammatory and infectious diseases.

*P. amboinicus* ethanolic extract inhibited the expression of ICAM-1, VCAM-1, and CD40 in obese rats, in addition to decreasing the levels of oxidative stress and inflammatory markers [58]. *P*. *amboinicus* also showed diuretic effects associated with improved electrolyte balance [54]. These results emphasize the effectiveness of this species in metabolic diseases such as hypertension and diabetes, which stand out as public health problems.

Regarding the analgesic activity, it was observed [53] that the aqueous extract decreased the writhing response and dose dependently inhibited formalin-induced paw-licking behavior in the late phase. Lopes et al. [54] showed that the alcoholic, hydroalcoholic, and aqueous extracts also showed analgesic effects by decreasing the percentage of abdominal contortions in mice, with the alcoholic extract showing the most significant effects.

The anti-inflammatory activity of *P*. *amboinicus* was also observed through the membrane stabilization method (HRBC) by [61], who demonstrated that the aqueous extract of the leaves (500 µg/mL) showed results comparable to hydrocortisone sodium [61]. Another species of the genus, *P. hadiensis*, was found to inhibit platelet and promote membrane stabilization in HRBC [65]. The terpenoid fraction of *P. hadiensis* presented excellent radical-scavenging activity [66], while the diethyl ether and n-hexane extracts of the leaves inhibited COX-2, demonstrating that the species has anti-inflammatory and antioxidant activities [67].

The study of [62] demonstrated the antiviral, anti-inflammatory, and antioxidant effects of the ethanol extract of *P. barbatus* against HIV-1. The extract inhibited the production of pro-inflammatory cytokines and reduced the expression of HIV-1 reverse transcriptase (CI_50_ = 62.0 μg/mL). In addition, the extract showed a relevant antioxidant effect. However, the mechanisms underlying these actions remain to be determined.

A study by [63] evaluated the anti-inflammatory, antimicrobial, and antioxidant activities of *P. caninus* essential oil, demonstrating that 200 and 300 mg/kg doses significantly inhibited the late phase of carrageenan-induced paw edema. The essential oil also demonstrated significant activity against a broad spectrum of pathogens, including Gram-positive and Gram-negative bacteria and some fungal strains. Moreover, the extract presented a concentration-dependent DPPH-scavenging activity with an EC_50_ value of 3.5 μL/mL, indicating significant antioxidant activity in vitro. These effects are possibly mediated by camphor (22.36%) and α-thujene (14.48%), the significant components in the essential oil.

Concerning other species listed in this review, the ethanolic and cyclohexane extracts of *P. forsteri* were found to reduce the levels of IL-6 and TNF-α, demonstrating promising in vitro anti-inflammatory activity in LPD-stimulated THP-1 cells [64]; The hydroalcoholic extract of *P. neochilus* showed healing effects associated with skin reepithelialization marked by the presence of fibroblasts, collagen fibers, and blood vessels in scars of Wistar rats [68]; Different extracts of *P. scutellarioides* [69] inhibited NO production, indicating inhibition of macrophage activation; *P. zeylanicus* extracts inhibited 5-LOX expression in stimulated human neutrophils but failed to show free radical scavenging activity and inhibit ROS production [70]. The dichloromethane extract (DCM) of this species showed significant antibacterial activity against methicillin-resistant *Staphylococcus aureus* with a minimum inhibitory concentration (MIC) of 62.5 g/mL [36,70]. These findings point to the pharmacological potential of *Plectranthus* species in acute and chronic inflammation and infection.

### 2.4. Methodological Quality/Risk of Bias Analysis

The methodological quality assessment/risk of bias analysis was performed for in vivo studies. For the first question regarding appropriate allocation, only the study by [53] was classified as having a high risk of bias for an inability to assess the risk and design characteristics of the groups. In contrast, the other studies were given a low risk. Regarding blind group allocations during the experiments, only the study in [56] reported accurate information, presenting a low risk, while the studies in [53,55,57,58,60,68] obtained an unclear risk (Table 4).

Nine studies were clear about animal allocation during the experimental period: those in [52,54,55,56,57,58,60,63,68]. Only the study in [53] presented a high risk for this item. In the blinding before the animal intervention stage, the study by [52] was the only one that reported performing this step. The studies in [53,55,56] had a high risk for this question, while [56,57,58,60,63,68] had an unclear risk of bias.

Question 9 asked if the animals were randomly selected for the result evaluation. Most studies (six) had an unclear risk regarding random animal evaluation (Chang et al., 2010 [52]; Duraisamy et al., 2021 [57]; El-Hawary et al., 2012 [54]; Gurgel et al., 2009 [55]; Harefa et al., 2021 [58]; Hsu et al., 2011 [56]; Manjamalai et al., 2012 [60]; Rêgo et al., 2021 [68]; Tadesse et al., 2011 [63]). Only the study in [53] presented information on the blinded evaluation outcome reported.

For question 8, most authors did not present data to classify the risk, with [52,55,57,58,60,63,68] obtaining an unclear risk of bias and [56] presenting a low risk.

**Figure 3:**
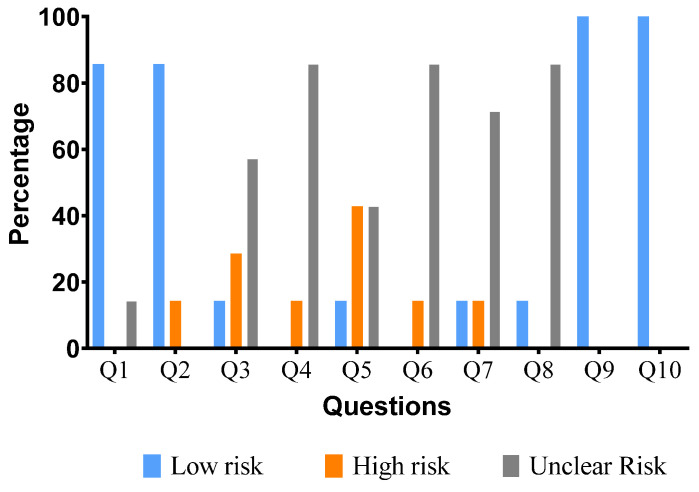
Percentage of the methodological quality evaluation results from the articles concerning the ten items.

Lastly, for items 9 (selective data results) and 10 (other sources of bias), all studies presented a low risk, with the percentage of the different types of bias expressed in Figure 3.

## 3. Materials and Methods

### 3.1. Review Outline and Data Selection, Procedure, and Analysis

The present study is a descriptive systematic literature review (SR) developed according to the PRISMA guidelines [73]. Given the objective of this study, five guiding questions were elaborated: Which species from the *Plectranthus* genus are described for treating inflammation and pain? What signs and symptoms are portrayed in the studies related to inflammation or pain? Are there species involved in the treatment of both conditions? Of the species found in ethnobotanical survey studies, have pharmacological tests been performed to investigate their anti-inflammatory or analgesic/antinociceptive activities? What are the characteristics of the studies found, and what are the biases they present?

The articles were collected from PubMed (Central: PMC- National Library of Medicine National Institutes of Health), ScienceDirect (Elsevier), SciVerse Scopus, and Web of Science (Main Collection—Clarivate Analytics) from December 2006 to April 2023. A total of fourteen different combinations using English descriptors were adopted in the search. Table S1 shows the details from the accessions, broken down by research category (*Plectranthus* and inflammation and *Plectranthus* and pain/nociception).

The selection criteria included fully available papers published in any language. Studies that did not contain the correct species specification, those that presented the description of the plant’s use only indicating the body or organ system, and other reviews were excluded. The relative frequency of citation (RFC) of each species is calculated by the number of works mentioning the use of species divided by the total number of works.

Two researchers (M.O.B. and G.M.d.L.L.) conducted the search with no articulation that could influence data collection. During the screening, an eligibility parameter form was applied to evaluate the titles and abstracts from the findings. Following this initial step, a detailed reading of the studies to confirm their inclusion or exclusion. Subsequently, the results from the two investigators were compared, and any divergences were resolved. A consensus between the parties determined the final sample and the data extraction step commenced.

### 3.2. Review Outline and Data Selection Procedure

The selected studies were classified into ethnobotanical surveys and pharmacological trials. Data extraction was performed following the PICOT (P—population, I—intervened, C—control, O—outcome, and T—type of study) process, adapted to each research nature.

Thus, in ethnobotanical surveys, the highlighted information concerned the: research place, country, cited species, the local name mentioned, an indication of use related to pain, the indication of use related to inflammation, a form of use, method of preparation, and conduct of the use. The pharmacological assays had the following elements extracted: study objective, studied species, type of study, chosen animal model, performed protocols, tested botanical form, plant part used, identified active principle, and results.

In addition, methodological quality assessment tools were adopted. SYRCLE RoB was used for pharmacological studies with non-human animals (in vivo and ex vivo) [74]. Based on Cochrane Collaboration’s criteria, SYRCLE’s RoB contains 10 entries, which fall into 6 types of bias: selection bias; performance bias; detection bias; attrition bias; reporting bias, and other biases [75].

The items considered by Cochrane Collaboration are randomization, allocation, blinding, data from incomplete outcomes, and funding source bias [74]. Thus, after carefully examining each study, the results were classified as “low risk of bias”, “high risk of bias”, and “unclear risk of bias”. It is noteworthy that in vitro preclinical and chemical research were not, at this time, considered since there are no validated instruments to examine their quality [76].

## 4. Conclusions

The present review contributed to the identification of different *Plectranthus* species investigated or used in the treatment of inflammatory and painful conditions. Most studies in the review consisted of ethnobotanical surveys highlighting their relevance to drug development research.

*Plectranthus amboinicus* presented the highest prevalence among studies, confirming the species’ ethnobotanical and pharmacological importance, especially in inflammatory, infectious, and metabolic diseases. In this context, carvacrol and rosmarinic acid, secondary metabolites identified in extracts and essential oils of this species, are promising drug candidates.

The species Plectranthus aliciae, Plectranthus barbatus, Plectranthus caninus, Plectranthus forsteri, Plectranthus hadiensis, Plectranthus neochilus, Plectranthus scutellarioides, and Plectranthus zeylanicus also showed relevant pharmacological activities such as antiviral, antioxidant, antimicrobial, antifungal and anti-inflammatory and as such are of interest in pharmacological research.

While several classes of secondary compounds have been isolated and characterized, their individual and relative contributions to the pharmacological effects of each species need to be better investigated. This approach will significantly contribute to elucidating the mechanisms underlying the effects of action and signaling pathways in the pathogenesis of *Plectranthus* species in inflammatory and painful responses.

## Figures and Tables

**Table 2:** Indications and relative frequency of citation (RFC) of *Plectranthus* species.

Species	Number of Citations	RFC	Use in Inflammation	Use in Pain Conditions
*Plectranthus amboinicus* (Lour.) Spreng.	9	0.23	Wound healing, fever, gastritis, bronchitis, uterine inflammation, inflammation of internal organs, nonspecified inflammation, abscess, asthma	Headache, sore throat, local pain, nonspecified pain
*Plectranthus barbatus* (Andrews) Benth.	8	0.20	Fever, gastritis, hepatitis, labyrinthitis	Heartburn, menstrual cramps, headache, toothache, stomachache, nonspecific pain, local pain, recurrent pain, migraine
*Plectranthus neochilus* Schltr. Schtr.	2	0.05	Labyrinthitis	Cramps, pain
*Plectranthus coleoides* Benth.	1	0.03	Wound healing	Labor pain
*Plectranthus kilimandschari* (Gürke ex Engl.) H.I.Maass Gurke	1	0.03	-	Chest pain
*Plectranthus lanuginosus* (Hochst. ex Benth.) Agnew	1	0.03	Tonsillitis	-
*Plectranthus ornatus* Codd.	2	0.05	Gastritis	Bellyache, headache
*Plectranthus rugosus* Wall. ex Benth.	1	0.03	Wound healing	-
*Plectranthus scutellarioides* (L.) R. Br.	1	0.03	-	Headache
*Plectranthus zeylanicus* Benth.	1	0.03	Fever	-

**Table 3:** Main aspects of pharmacological assays.

Species	Author Year	Objective of the Study	Type of Study	Protocol Used	Type of Extract	Part of the Plant Used	Chemical Component	Findings
*P. amboinicus* (Lour.) Spreng	Gurgel et al., 2009 [55]	Evaluate the anti-inflammatory and antitumor activities.	In vivo	Carrageenan-induced paw edema, sarcoma-180 and Erlich’s ascites carcinoma cancer models.	Hydroalcoholic extract	Leaves	NR	Animals treated with the extract showed a significant decrease in edema at 150, 250, and 350 mg/kg (i.p.). The extract also inhibited the growth of sarcoma-180 and Ehrlich’s ascites tumors.
*P. amboinicus* (Lour.) Spreng	Ravikumar et al., 2009 [61]	Evaluate the anti-inflammatory activity.	In vitro	Human red blood cell (HRBC) membrane stabilization assay.	Aqueous extract	Leaves	NR	The extract (500 µg/mL) showed significant anti-inflammatory activity, comparable to hydrocortisone sodium.
*P. amboinicus* (Lour.) Spreng	Chang et al., 2010 [52]	Investigate the anti-inflammatory activity in a rheumatoid arthritis (RA) model.	In vivo	In collagen-induced arthritis (ASD) model, the following parameters were evaluated: serum levels of anti-collagen IgG, IgM, and C-reactive protein (CRP), concentrations of TNF-α, IL-6, and IL-1β production in peritoneal exudate (PEC) cells.	Aqueous extract	Whole plant	NR	The plant extract treatment significantly inhibited paw swelling and arthritis symptoms. Rats treated with the highest extract dose presented significantly reduced IgM, CRP, TNF-α, IL-6, and IL-1β levels.
*P. amboinicus* (Lour.) Spreng	Hsu et al., 2011 [56]	Investigate the effects on osteoclastogenesis and inflammatory bone erosion in mice in a collagen-induced arthritis model. Identify the active component of the plant involved in the regulation of osteoclastogenesis.	In vitro and in vivo	Cell viability was determined in an MTT assay. Bone marrow macrophages and RAW 264.7 had the expression transcriptional factors analyzed by immunofluorescence, collagen-induced arthritis in mice was assessed through the quantification of IL-1ß and TNF-α, analysis of arthritic index, paw thickness, and histopathological evaluation.	Crude extract	Leaves	Rosmarinic acid	*P. amboinicus* significantly inhibited bone resorption by mature osteoclasts. Rosmarinic acid showed potent inhibition of NF-κB NFATc1 in RANKL-stimulated BMM and inhibited RANKL-induced formation of TRAP-positive multinucleated cells.
*P. amboinicus* (Lour.) Spreng	Chiu et al., 2012 [53]	Investigate the analgesic and anti-inflammatory properties of the aqueous extract and essential oil.	In vivo and in vitro	The analgesic effect was evaluated in acetic acid and formalin models. The anti-inflammatory activity was assessed in carrageenan-induced paw edema by evaluating oxidative stress, cytokine production, and protein expression in tissue homogenates and cultures of LPS-stimulated RAW 264.7 cells.	Aqueous extract and essential oil	Whole plant	Carvacrol, thymol, α-humulene, undecanal, C-terpinene, R-cymene, caryophyllene oxide, α-terpineol, and β-seline	The reduced abdominal contortions and paw-licking behavior demonstrated analgesic activity. The anti-inflammatory effect was due to the modulation of antioxidant enzymes in the liver and decreased levels of malondialdehyde (MDA), TNF-α, and cyclooxygenase 2 (COX-2). In vitro, the treatment inhibited cytokine production and prevented NF-κB activation.
*P. amboinicus* (Lour.) Spreng	El-Hawary et al., 2012 [54]	Investigate the phenolic content and evaluate the antioxidant, anti-inflammatory, analgesic, diuretic, cytotoxic, and antimicrobial activities.	In vivo and in vitro	Chemical analysis was performed through UPLC–MS. In vivo testing evaluated LD50, blood glutathione levels in diabetic animals, carrageenan-induced paw edema, contortions induced by acetic acid, and diuretic effect. The antimicrobial effect was determined through the agar diffusion method.	Alcoholic, aqueous, and hydroalcoholic extracts	Stem, leaves, and roots	Caffeic acid, rosmarinic acid, coumaric acid, crosseriol, stem, luteolin, quercithin, and eryodithiol	The different extracts showed antioxidant, anti-inflammatory, analgesic, diuretic, cytotoxic, and antimicrobial activities, although their potency varied significantly.
*P. amboinicus* (Lour.) Spreng	Manjamalai et al., 2012 [60]	Evaluate the antimicrobial and anti-inflammatory activity.	In vivo and in vitro	The antimicrobial activity was evaluated through the determination of the minimum inhibitory concentration (MIC) and minimum fungicidal concentration (MFC), the anti-inflammatory activity was assessed in xylene-induced ear edema, carrageenan-induced paw edema, and ovalbumin-induced allergic inflammation.	Essential oil	Leaves	Carvacrol, thymol, cys-caryophyllene, t-caryophyllene, and p-cymene	The essential oil had promising antimicrobial effects against bacteria and fungi and inhibited the inflammatory response triggered by different harmful stimuli.
*P. amboinicus* (Lour.) Spreng	Chen et al., 2014 [40]	Identify the constituents and evaluate the anti-inflammatory effect. Prepare analogs to maximize the anti-inflammatory effect.	In vitro	The AP-1 binding affinity in TPA-treated HeLa cells and TNF-α expression by LPS-stimulated human histiocytic lymphoma U-937 cells were evaluated through an isolation-guided bioassay. The cytotoxicity of the human fibroblast cell line Detroit551 cells was determined in an MTT assay.	Hexane and aqueous extracts	Leaves and stem	1-2-(3,4-dihydroxybenzylidenyl)-3-(3,4-dihydroxyphenyl)-4-hydroxypentanedioic acid, shimobashiric acid, salvianolic acid, rosmarinic acid (2-alkylidenenyl-4-cyclopentene-1), 3-diones), thymoquinone	*P. amboinicus* extract inhibited the binding of AP-1 to its consensual DNA sequence. Tymquinone, isolated from the hexane extract, suppressed TNF-α expression, indicating in vitro anti-inflammatory activity.
*P. amboinicus* (Lour.) Spreng	Leu et al., 2019 [59]	Analyze the anti-inflammatory mechanism of compounds extracted from *P. amboinicus* in the NLRP3 inflammasome signaling pathway.	In vitro	Phorbol-12-myristate with 13-acetate (PMA)-differentiated and LPS-stimulated THP-1 monocytic leukemia cells were used to examine the effect of PA-F4, a *P. amboinicus* extract, on the inflammasome signaling pathway.	NR	NR	Rosmarinic acid, cirsimaritin, salvigenin, carvacrol	PA-F4 inhibited ASC oligomerization, KC efflux, the caspase-1/ IL-1b/ IL-18 release reaction, and NF-kB p65 activation, demonstrating an interference with NLRP3- NF-kB signaling pathway in LPS-activated macrophages.
*P. amboinicus* (Lour.) Spreng	Duraisamy et al., 2021 [57]	Evaluate the anti-inflammatory activities.	In vivo and in vitro	The analgesic and anti-inflammatory effect was analyzed in the formalin test and correlated with the analysis of inflammatory mediator production and protein expression in vitro.	Aqueous and ethyl acetate extracts	Leaves	Carbohydrates, steroids, flavonoids, saponins, glycosides, terpenoids	The extracts inhibited nociceptive responses and the paw edema through the modulation of the inflammatory reaction, which was associated with a decrease in oxidative stress markers and inhibition of gene expression of iNOS, COX-2, IL-1β, histamine receptor 1, and NF-κB. In addition, P. *amboinicus* inhibited NO production by in vitro-stimulated macrophages.
*P. amboinicus* (Lour.) Spreng	Harefa et al., 2021 [58]	Analyze the effects of the treatment on ICAM-1, VCAM-1, and CD40 expression in obese rats.	In vivo	Obesity was induced in Wistar rats through a standard diet of CP511 with the addition of a high-fat diet for 21 weeks. The expression of ICAM-1 and VCAM-1 in the plasma was analyzed by ELISA, while Immunohistochemistry was used to analyze CD40 expression in the aorta.	Ethanol extract	Leaves	NR	The treatment showed a mild decrease in ICAM-1 and VCAM-1 levels in the blood plasma. The same occurred with the expression of CD40 in the intimal layer of the aorta of treated rats.
*P. barbatus* Andrews	Kapewangolo et al., 2013 [62]	Investigate the antioxidant, anti-inflammatory, and anti-HIV-1 activities of the species.	In vitro	The anti-HIV-1 activity was assessed through inhibition of protease (PR) and reverse transcriptase (RT), cytotoxicity was evaluated in peripheral blood mononuclear cells (SPMC) and TZM cells, free radical-scavenging activity was used to assess antioxidant activity, while cytometric matrix Th1/Th2/Th17 cytokine production was used to determine the anti-inflammatory activity.	Ethanol extract	Leaves	NR	The extract inhibited HIV-1 PR with a CI_50_ of 62.0 μg/mL and induced cell proliferation in HIV-positive and HIV-negative cells. Finally, the extract showed a relevant antioxidant effect (CI_50_ = 16 μg/mL) and reduced the production of pro-inflammatory cytokines.
*P. caninus* Roth	Tadesse et al., 2011 [63]	Characterize the chemical composition of *P. caninus* essential oil and investigate its antioxidant and anti-inflammatory activities.	In vivo and in vitro	Gas chromatography coupled to mass spectrometer (GC–MS), determination of the minimum inhibitory concentration (MIC), antioxidant activity by the 2,2-diphenyl-1-picrylhydrazi DPPH method, carrageenan-induced paw edema.	Essential oil	NR	Camphor and α-thurjene	The essential oil showed significant activity against a broad spectrum of pathogens, including Gram-positive and Gram-negative bacteria and some fungal strains. The extract showed a concentration-dependent DPPH-scavenging activity and inhibited paw edema in the late phase of inflammation.
*P. forsteri* Benth.	Nicolas et al., 2023 [64]	Investigate the chemical composition and anti-inflammatory potential of *P. forsteri*.	In vitro	Primary culture of human monocyte-derived macrophages THP-1 cells were assessed for cytotoxicity and production of TNF-α, IL-6, IL-10, and IL-1β.	Ethanolic (ePE) and cyclohexane (cPE) extract of *C. forsteri*	Whole plant	Coleon U (1), coleon U-quinone (2), 8α,9α-epoxycoleon U-uinone (3), 7α- hydroxyroyleanone (4), 6β,7α-dihydroxyroyleanone (5), 7α-acetoxy-6β- hydroxyroyleanone (6) and 7α-formyloxy-6β-hydroxyroyleanone (7)	Both extracts significantly inhibited cytokine production in LPS-stimulated THP-1 cells and human macrophages.
*P. hadiensis* (Forssk.) Schweinf. ex Sprenger	Menon et al., 2011 [65]	Investigate possible anti-inflammatory and cytotoxic activities.	In vitro	Analysis of ADP-induced platelet aggregation, human red blood cell (HRBC) membrane stabilization assay, MTT cytotoxicity test.	Methanolic extract	NR	NR	The extract significantly inhibited platelet aggregation and promoted membrane stabilization of HRBC, which was comparable in magnitude to the standard drug diclofenac.
*P. hadiensis* (Forssk.) Schweinf. ex Sprenger	Menon et al., 2014 [66]	Investigate the antioxidant and anti-inflammatory activities of the terpenoid fraction isolated from *P. hadiensis.*	In vitro	DPPH assay for antioxidant activity, reduction capacity by the potassium ferricyanide reduction method, nitric oxide elimination capacity, evaluation of bovine serum albumin, stabilization of the erythroblast membrane of red blood cells.	Aqueous and ethanol extract	NR	NR	The terpenoid fraction exhibited significant free radical-scavenging activity.
*P. hadiensis* (Forssk.) Schweinf. ex Sprenger	Schultz et al., 2021 [67]	Investigate the anti-inflammatory, antioxidant, and antibacterial activities.	In vitro	In vitro screening for the selective inhibition of COX-2, COX-1, and 15-LOX. Evaluation of the growth of multidrug-resistant *Staphylococcus aureus*, *Listeria innocua*, *Listeria monocytogenes*, and *Escherichia coli* K12, DPPH assay for antioxidant activity, and determination of total phenolic content (TCT).	Diethyl ether extract	Leaves	NR	Nine extracts, including the ethyl ether extract of *P. hadiensis*, were active as COX-2 inhibitors (IC_50_ < 20 g/mL). There was no counteractivity between the inhibition of COX-2 and 15-LOX in these extracts. No relevant activity was observed regarding the other analysis.
*Plectranthus madagascariensis* var. aliciae Codd	Lambrechts et al., 2022 [51]	Investigate the antibacterial effects and healing potential of gold nanoparticle-encapsulated *P. aliciae* extract, compound rosmarinic acid (AuNPRA), and tetracycline (AuNPTET).	In vitro	The antibacterial activity of nanoparticles was tested against *C. Acnes* (ATCC^®^ 6919), *S. epidermidis* (ATCC^®^ 35984), and a combination of *C. acnes* and *S. epidermidis* under anaerobic and aerobic growth conditions. The cytotoxicity and wound healing potential were also evaluated using human keratinocytes (HaCaT).	Ethanolic	Leaves	Rosmarinic acid + gold nanoparticles	None of the nanoparticles presented antibacterial or antibiofilm against *C. acnes* and *S. epidermis*. However, they showed significant wound healing potential. Rosmarinic acid showed effectiveness at the highest concentration (500 g/mL).
*P. neochilus* Schltr.	Rêgo et al., 2021 [68]	Investigate the effects of a gel formulation containing the combination of *P. neochilus* and *Cnidoscolus quercifolius* in tissue repair in rat skin wounds.	In vivo	Tissue repair in skin wounds of rats.	Hydroalcoholic extract	Slum Bark	NR	The macroscopic evaluation revealed angiogenic potential. The histomorphometry of the skin revealed reepithelialization of the epidermis and superficial dermis with longitudinal collagen fibers, fibroblasts, and blood vessels. The deeper dermis was marked with transverse and longitudinal collagen fibers, blood vessels, and inflammatory cells.
*P. scutellarioides* (L.) R.Br.	Fakhriati et al., 2018 [69]	Determine the anti-inflammatory activity.	In vitro	Nitrite quantification through the Griess method.	Ethanolic, ethyl acetate, and aqueous extract	Leaves	NR	The ethanol extract showed the most potent inhibitory effect on nitrite production by macrophages.
*P. zeylanicus* Benth.	Napagoda et al., 2014 [70]	Investigate the effects on 5-LOX activity and free radical scavenging by *P. zeylanicus* extracts and analyze their chemical constituents.	In vitro	Evaluation of bioactivity: 5-lipoxygenase (5-LOX) activity in intact neutrophils and whole blood, activity of 5-LOX in cell-free assays using semipurified 5-LOX, DPPH assay for antioxidant activity, measurement of reactive oxygen species in neutrophils. Phytochemical screening: Bioassay-guided fractionation, Liquid chromatography coupled to mass spectrometry analysis, gas chromatography coupled to mass spectrometry analysis.	Hexane, dichloromethane, ethyl, and methanolic extracts	Whole plant	Coleone P, cinasassiol A and C and caloric acid	Regarding the pharmacological activities, the hexane and dichloromethane extracts of *P. zeylanicus* showed a suppressive effect of 5-LOX in stimulated human neutrophils and the recombinant human isolate of 5-LOX.
*P. zeylanicus* Benth.	Napagoda et al., 2022 [71]	Evaluate the antimicrobial activity of different extracts of *P. zeylanicus* and characterize bioactive secondary metabolites.	In vitro	The antibacterial activity of the purified compound against *S. aureus*, *S. saprophyticus*, *E. faecalis*, *S. typhi*, *P. aeruginosa*, and nine clinical isolates of methicillin-resistant S. aureus was determined by the broth microdilution method, cell-based 5-LOX activity assay	n-hexane, dichloromethane (DCM), ethyl acetate (EtOAc), and methanolic	NR	7-acetoxy-6-hydroxyroyleanone	The dichloromethane extract (DCM) showed a potent effect against several bacterial species. The isolated compound showed strong antibacterial activity against methicillin-resistant *Staphylococcus aureus* and inhibited 5-LOX in free and cell-based assays.

NR—Information not available on paper.

**Table 4:** Methodological quality for preclinical pharmacological trials per reviewer.

Reference	Q1	Q2	Q3	Q4	Q5	Q6	Q7	Q8	Q9	Q10
Gurgel et al., 2009 [55]	+	+	?	?	-	?	-	?	+	+
Chang et al., 2010 [52]	+	+	-	?	+	?	+	?	+	+
Hsu et al., 2011 [56]	+	+	+	?	?	?	?	+	+	+
Tadesse et al., 2011 [63]	+	+	?	?	?	?	?	?	+	+
Chiu et al., 2012 [53]	?	-	?	-	-	-	?	?	+	+
El-Hawary et al., 2012 [54]	+	+	-	?	-	?	?	?	+	+
Manjamalai et al., 2012 [60]	+	+	?	?	?	?	?	?	+	+
Duraisamy et al., 2021 [57]	+	+	?	?	?	?	?	?	+	+
Label:										
Yes	+	Low risk of bias								
No	-	high risk of bias								
Not clear	?	The risk of bias is not clear								

Q = Question. Q1—Was the allocation sequence adequately generated and applied? Q2—Were the groups similar at baseline, or were they adjusted for confounding in the analysis? Q3—Was a blind allocation of the different groups performed adequately during the test? Q4—Were the animals randomly housed during the experiment? Q5—Were the technicians and researchers blinded by the intervention each animal received during the experiment? Q6—Were animals randomly selected for the result assessment? Q7—Was the advisor blind from the results? Q8—Were incomplete results adequately treated? Q9—Are study reports free from selective report outcomes? Q10—Was the study free of other problems that could result in a high risk of bias?

## Supplementary Materials

The following supporting information can be downloaded at: https://www.mdpi.com/article/10.3390/molecules28155653/s1, Table S1. Outline of the descriptor applications and their combinations to databases. Table S2. Description of initial and final results by accessed database.

## Sample Availability

Samples of the compounds are available from the authors.
