# Supplementary material for: Plectranthus Species with Anti-Inflammatory and Analgesic Potential: A Systematic Review on Ethnobotanical and Pharmacological Findings
Source: Molecules. 2023 Jul 26;28(15):5653. doi: 10.3390/molecules28155653 (PMC10419981; doi:10.3390/molecules28155653)
Supplement: Supplementary file 1 [file molecules-28-05653-s001.zip › molecules-2416633-supplementary.pdf]

Supplementary data

Plectranthus Species with Anti-Inflammatory and Analgesic Potential: A Systematic Review on Ethnobotanical and Pharmacological Findings

Maysa de Oliveira Barbosa [1,†], Polrat Wilairatana [2,*,†], Giovana Mendes de Lacerda Leite [1], Gyllyandeson de Araújo Delmondes [3], Lucas Yure Santos da Silva [1], Silvio Caetano Alves Júnior [4], Lindaiane Bezerra Rodrigues Dantas [1], Daniel Souza Bezerra [1], Izabel Cristina Santiago Lemos de Beltrão [1], Diógenes de Queiroz Dias [1], Jaime Ribeiro-Filho [4], Cícero Francisco Bezerra Felipe [5], Henrique Douglas Melo Coutinho [1], Irwin Rose Alencar de Menezes [1,*,†] and Marta Regina Kerntopf Mendonça [1]

1 Department of Biological Chemistry, Regional University of Cariri-URCA, Crato 63105-000, CE, Brazil; maysabarbosa.ce@gmail.com (M.d.O.B.); giovanalacerda_@hotmail.com (G.M.d.L.L.); lucas.yure@urca.br (L.Y.S.d.S.); lindaianebrd@gmail.com (L.B.R.D.); danielbezerra02@gmail.com (D.S.B.); izabel.lemos@urca.br (I.C.S.L.d.B.); diogenes@gmail.com (D.d.Q.D.); hdmcoutinho@gmail.com (H.D.M.C.); martaluiz@yahoo.com.br (M.R.K.M.)
2 Department of Clinical Tropical Medicine, Faculty of Tropical Medicine, Mahidol University, Bangkok 10400, Thailand
3 Collegiate of Nursing, Federal University of Vale do São Francisco (UNIVASF), Petrolina 56304-917, PE, Brazil; gyllyandeson.delmondes@univasf.edu.br
4 Oswaldo Cruz Foundation (FIOCRUZ), Fiocruz Ceará, Eusébio 61773-270, CE, Brazil; silvio.alves.bio@gmail.com (S.C.A.J.); jaime.ribeiro@fiocruz.br (J.R.-F.)
5 Department of Molecular Biology, Federal University of Paraiba, João Pessoa 58051-900, PB, Brazil; cicero@dbm.ufpb.br
* Correspondence: polrat.wil@mahidol.ac.th (P.W.); irwin.alencar@urca.br (I.R.A.d.M.); Tel.: +55-88-31021291 (I.R.A.d.M.)
† These authors contributed equally to this work.

Table S1. Outline of the descriptor applications and their combinations to databases.

Plectranthus AND PAIN/NOCICEPTION				
**Combination**	**Translation of the search**			
	**PubMed**	**ScienceDirect**	**Scopus**	**Web of Science**
Plectranthus and analgesic effect	("*Plectranthus*"[MeSH Terms] OR "*Plectranthus*"[All Fields]) AND ("Analgesics"[All Fields] OR "Analgesics"[MeSH Terms] OR "Analgesics"[All Fields] OR "Analgesic"[All Fields]) AND Effect[All Fields]	NR	(TITLE-ABS KEY(*Plectranthus* AND Analgesic effect))	(*Plectranthus* AND Analgesic effect)
Plectranthus and antinociceptive effect	("*Plectranthus*"[MeSH Terms] OR "*Plectranthus*"[All Fields]) AND Antinociceptive[All Fields] AND Effect[All Fields]	NR	(TITLE-ABS-KEY(*Plectranthus* AND Antinociceptive effect))	(*Plectranthus* AND Antinociceptive effect)
Plectranthus and nociception	("*Plectranthus*"[MeSH Terms] OR "*Plectranthus*"[All Fields]) AND ("Nociception"[MeSH Terms] OR "Nociception"[All Fields])	NR	(TITLE-ABS-KEY(*Plectranthus* AND Nociception))	(*Plectranthus* AND Nociception)

Plectranthus and pain	("*Plectranthus*"[MeSH Terms] OR "*Plectranthus*"[All Fields]) AND ("Pain"[MeSH Terms] OR "Pain"[All Fields])	NR	(TITLE-ABS-KEY(*Plectranthus* AND Pain))	(*Plectranthus* AND Pain)
Plectranthus and pain and ethnopharmacology	("*Plectranthus*"[MeSH Terms] OR "*Plectranthus*"[All Fields]) AND ("Pain"[MeSH Terms] OR "Pain"[All Fields]) AND ("Ethnopharmacology"[MeSH Terms] OR "Ethnopharmacology"[All Fields])	NR	(TITLE-ABS-KEY(*Plectranthus* AND Pain AND Ethnopharmacology))	(*Plectranthus* AND Pain AND Ethnopharmacology)
Plectranthus and pain and ethnobotanical	("*Plectranthus*"[MeSH Terms] OR "*Plectranthus*"[All Fields]) AND ("Pain"[MeSH Terms] OR "Pain"[All Fields]) AND Ethnobotanical[All Fields]	NR	Not Applicable	Not Applicable
Plectranthus and pain and medicinal use	("*Plectranthus*"[MeSH Terms] OR "*Plectranthus*"[All Fields]) AND ("Pain"[MeSH Terms] OR "Pain"[All Fields]) AND Medicinal[All Fields]	NR	(TITLE-ABS-KEY(*Plectranthus* AND Pain AND Medicinal use))	(*Plectranthus* AND Pain AND Medicinal use)

***Plectranthus* AND INFLAMMATION**				
**Combination**	**Translation of the search**			
	**PubMed**	**ScienceDirect**	**Scopus**	**Web of Science**
Plectranthus and anti-inflammatory effect	("*Plectranthus*"[MeSH Terms] OR "*Plectranthus*"[All Fields]) AND ("Anti-inflammatory agents"[All Fields] OR "Anti-inflammatory agents"[MeSH Terms] OR ("Anti-inflammatory"[All Fields] AND "Agents"[All Fields]) OR "Anti-inflammatory agents"[All Fields] OR ("Anti"[All Fields] AND "Inflammatory"[All Fields]) OR "Antiinflammatory"[All Fields]) AND Effect[All Fields]	NR	TITLE-ABS-KEY(*Plectranthus* AND Anti-inflammatory effect)	(*Plectranthus* AND Anti-inflammatory effect)
Plectranthus and anti-inflammatory	("*Plectranthus*"[MeSH Terms] OR "*Plectranthus*"[All Fields]) AND ("Anti-inflammatory agents"[All Fields] OR "Anti-inflammatory agents"[MeSH Terms] OR ("Anti-inflammatory"[All Fields] AND "Agents"[All Fields]) OR "Anti-inflammatory agents"[All Fields] OR ("Anti"[All Fields] AND "Inflammatory"[All Fields]) OR "Antiinflammatory"[All Fields])	NR	TITLE-ABS-KEY(*Plectranthus* AND Anti-inflammatory)	*Plectranthus* AND Anti-inflammatory)
Plectranthus and anti-inflammatory and ethnopharmacology	("*Plectranthus*"[MeSH Terms] OR "*Plectranthus*"[All Fields]) AND ("Anti-inflammatory agents"[All Fields] OR "Anti-inflammatory agents"[MeSH Terms] OR ("Anti-inflammatory"[All Fields] AND	NR	TITLE-ABS-KEY(*Plectranthus* AND Anti-inflammatory AND Ethnopharmagology)	Not Applicable

	"Agents"[All Fields]) OR "Anti-inflammatory agents"[All Fields] OR ("Anti"[All Fields] AND "Inflammatory"[All Fields]) OR "Antiinflammatory"[All Fields]) AND ("Ethnopharmacology"[MeSH Terms] OR "Ethnopharmacology"[All Fields])			
Plectranthus and inflammatory	("*Plectranthus*"[MeSH Terms] OR "*Plectranthus*"[All Fields]) AND "Inflammatory" [All Fields]	NR	TITLE-ABS-KEY(*Plectranthus* AND Inflammatory)	(*Plectranthus* AND Inflammatory)
Plectranthus and inflammatory and ethnopharmacology	("*Plectranthus*"[MeSH Terms] OR "*Plectranthus*"[All Fields]) AND "Inflammatory" [All Fields] AND ("Ethnopharmacology"[MeSH Terms] OR "Ethnopharmacology"[All Fields])	NR	TITLE-ABS-KEY(*Plectranthus* AND Inflammatory AND Ethnopharmacolog y)	Not Applicable
Plectranthus and inflammatory and ethnobotanical	("*Plectranthus*"[MeSH Terms] OR "*Plectranthus*"[All Fields]) AND "Inflammatory" [All Fields] AND Etnobotanical[All Fields]	NR	Not Applicable	Not Applicable
Plectranthus and inflammatory and medicinal use	("*Plectranthus*"[MeSH Terms] OR "*Plectranthus*"[All Fields]) AND "Inflammatory" [All Fields] AND "Medicinal" [All Fields]	NR	TITLE-ABS-KEY(*Plectranthus* AND Inflammatory AND Medicinal use)	(*Plectranthus* AND Inflammatory AND Medicinal use)

NR= not reported.

Table S2. Description of initial and final results by accessed database.

Plectranthus and inflammation								
**Descriptors Combination**	**PubMed**		**Science Direct**		**Scopus**		**Web of Science**	
	**IR**	**FR**	**IR**	**FR**	**IR**	**FR**	**IR**	**FR**
Plectranthus and anti-inflammatory effect	205	9	240	73	34	14	14	4
Plectranthus and anti-inflammatory	229	14	387	184	49	19	20	5
Plectranthus and anti-inflammatory and ethnopharmacology	29	2	111	41	7	4	0	0
Plectranthus and inflammatory	234	12	212	8	70	29	27	7
Plectranthus and inflammatory and ethnopharmacology	29	1	113	42	5	4	0	0
Plectranthus and inflammatory and etnobotanical	2	2	0	0	2	1	0	0
Plectranthus and inflammatory and medicinal use	185	10	336	166	41	25	18	6
**Total by data bases**	**913**	**50** / **8***	**1399**	**514** / **9***	**208**	**96** / **21***	**79**	**22** / **7***
**Total**	**IR= 2.599**				**PR= 682; FR= 45***			
***Plectranthus* and pain/nociception**								
**Descriptors Combination**	**PubMed**		**Science Direct**		**Scopus**		**Web of Science**	
	**IR**	**FR**	**IR**	**FR**	**IR**	**FR**	**IR**	**FR**
Plectranthus and analgesic effect	78	9	154	75	8	4	5	1
Plectranthus and antinociceptive effect	41	3	56	27	2	1	1	0
Plectranthus and nociception	12	2	11	7	19	18	1	1

Plectranthus and pain	120	9	278	125	206	197	3	2
Plectranthus and pain and ethnopharmacology	31	4	136	48	147	135	1	1
Plectranthus and pain and ethnobotanical	77	8	55	52	57	50	0	0
Plectranthus and pain and medicinal use	105	8	256	109	188	167	1	1
**Total by data base**	**464**	**43 / 10***	**946**	**443 / 13***	**627**	**472 / 10***	**12**	**6 / 2***
**Total**		**IR= 2.049**				**PR= 964; FR= 35***		

IR= Initial Results. PR= Partial Result (after inclusion and exclusion criteria).FR= Final Result (selected after exclusion of duplicated articles).

**Species**	
Plectranthus amboinicus (Lour.) Spreng.	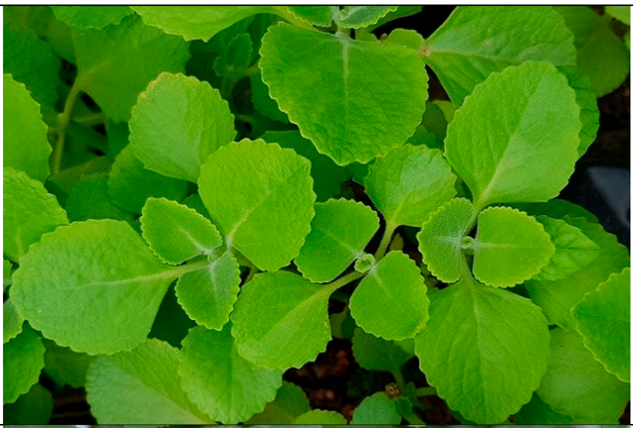
Plectranthus barbatus (Andrews) Benth.	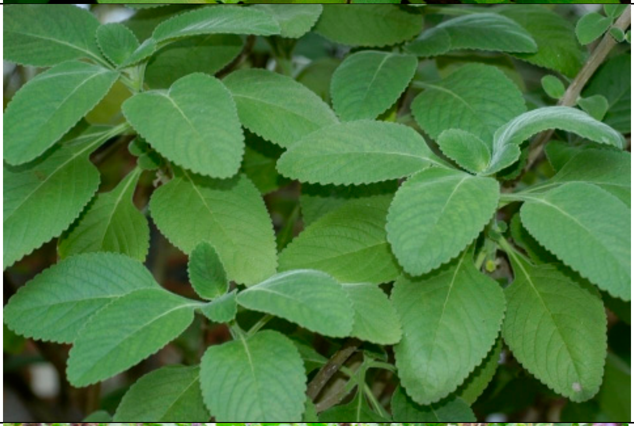
Plectranthus neochilus Schtr.	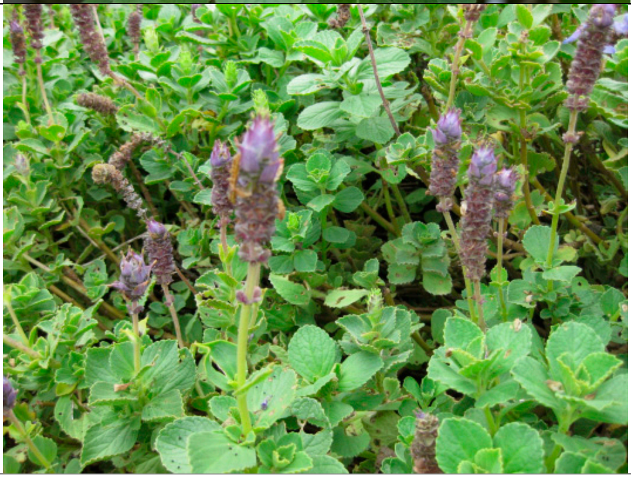

Species	
Plectranthus coleoides Benth.	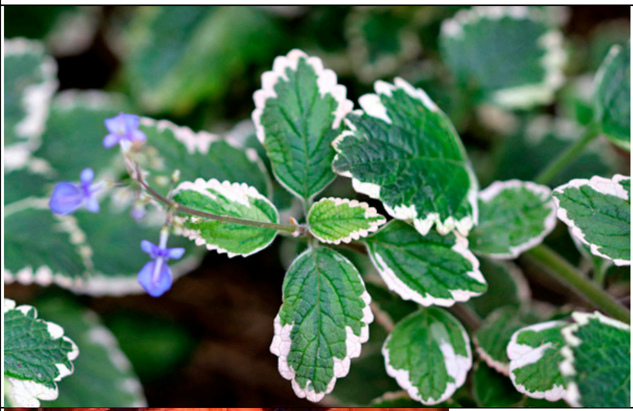
Plectranthus kilimandschari Gurke	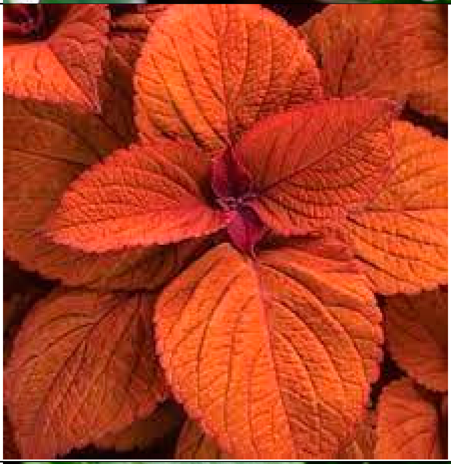
Plectranthus lanuginosus	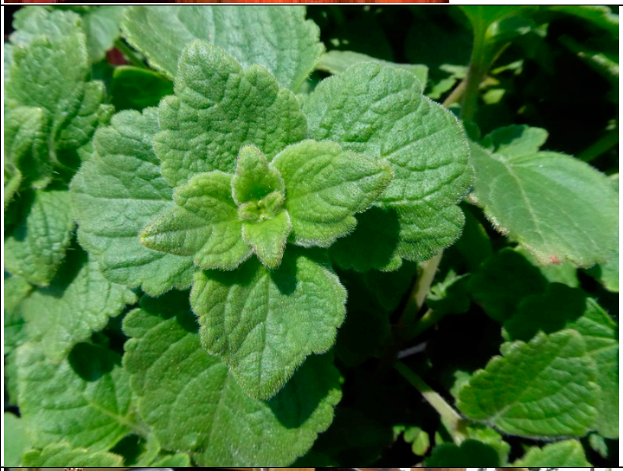
Plectranthus ornatus Codd.	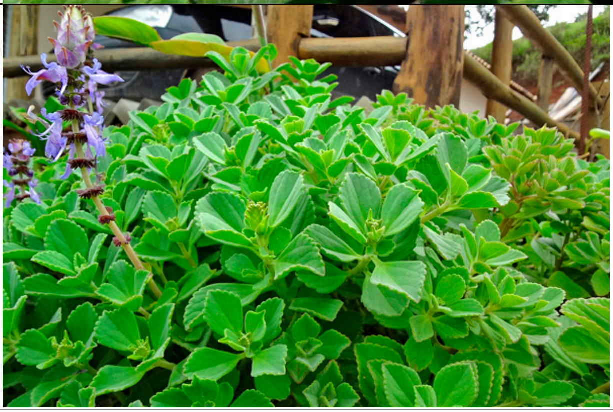

Species	
Plectranthus rugosus	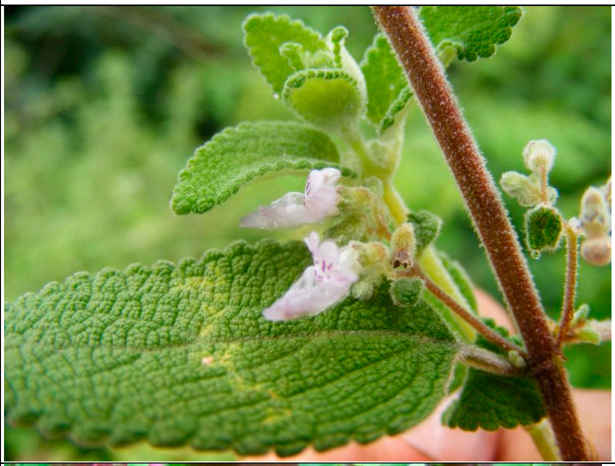
Plectranthus scutellarioides (L.) R. Br.	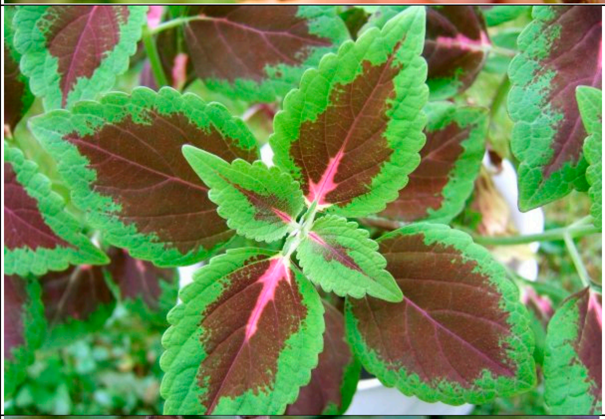
Plectranthus zeylanicus Benth.	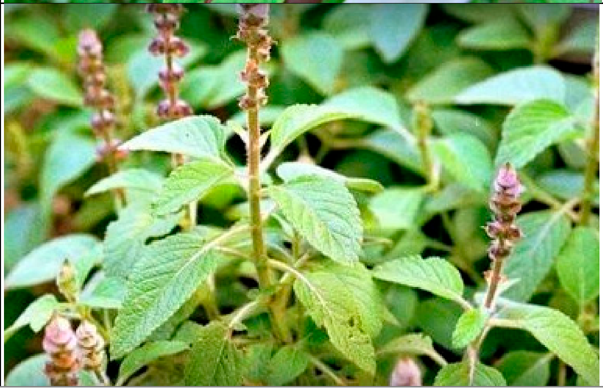
